# Vision Gaze-Driven Micro-Electro-Mechanical Systems Light Detection and Ranging Optimization

**DOI:** 10.34133/research.0756

**Published:** 2025-06-23

**Authors:** Shaotang Wei, Bo Gao, Junya Wang, Zheng You

**Affiliations:** School of Mechanical Science and Engineering, Huazhong University of Science and Technology, Wuhan 430074, China.

## Abstract

Micro-electro-mechanical systems (MEMS) light detection and ranging (LiDAR) systems are widely employed in diverse applications for their precise ranging and high-resolution imaging capabilities. However, conventional Lissajous scanning patterns, despite their design flexibility, are increasingly limited in meeting the growing demands for image quality. In this study, we propose a novel programmable scanning method that enhances angular resolution within defined regions of interest (ROIs). By applying parameter modulation techniques, we establish a direct, analytical link between the scanning trajectory and ROI placement, enabling precise resolution control. The proposed method increases point cloud density by 2 to 6 times across any ROI within a Lissajous scan, achieving localized improvements of up to 650%, independent of frequency constraints. Moreover, it reduces the design complexity of MEMS scanning mirrors by half, while maintaining comparable high-resolution performance. Incorporating a gaze-inspired trajectory modulation strategy and random modulation continuous wave ranging, we develop a MEMS LiDAR prototype that greatly enhances point cloud fidelity and enables accurate 3-dimensional imaging within ROIs—achieving a ranging accuracy of 2.4 cm (3σ). This approach not only improves angular resolution in targeted regions but also extends the practical applicability of MEMS LiDAR to multitarget tracking and recognition scenarios. Furthermore, the study establishes a robust theoretical framework for ROI-based trajectory control, contributing to the advancement of next-generation high-resolution imaging systems.

## Introduction

Micro-electro-mechanical systems scanning mirrors (MEMS-SM) have become pivotal in driving innovation in scanning imaging technologies [1] due to their high integration [2–4], rapid scanning capabilities [5,6], and programmable control features. These systems are widely employed in fields ranging from light detection and ranging (LiDAR) [7,8] and in vivo medical imaging [9–11] to projection displays [12]. MEMS-SM supports a variety of scanning trajectory designs such as Lissajous [13], Linescan [14], Rosette [15–17], and Raster [18,19], each offering unique advantages and limitations tailored to specific image resolution requirements.

For example, in Linescan applications, the slow axis typically employs triangular or sawtooth waveforms, which present a substantial challenge for high-speed operation [20,21]. This limitation can negatively impact the overall efficiency and resolution of the imaging system. In contrast, Rosette scanning is often effective only in imaging the central area, struggling to cover the entire field of view [22], thus limiting its applicability in scenarios that require comprehensive area coverage. On the other hand, Lissajous scanning, with its sinusoidal trajectory, is widely used across various optical imaging systems, such as microscopy and medical imaging [23,24], offering superior image performance compared to both Raster and Rosette trajectories [22,25,26]. Lissajous scanning is increasingly preferred in LiDAR systems [27] due to its ability to produce clean spectra that enable high-precision angular measurements [28,29]. This method is especially advantageous in MEMS-SM, as it supports all types of MEMS scanning mirrors, unlike Linescan, which is restricted to nonresonant configurations. Lissajous scanning allows for a balanced optimization of crucial performance metrics, making it ideal for applications like simultaneous localization and mapping (SLAM) and 3-dimensional (3D) imaging, where achieving high frame rates without compromising angular resolution is essential. As a result, Lissajous trajectories are an excellent choice for MEMS LiDAR systems.

The image resolution in Lissajous scanning modes is commonly assessed by calculating the fill factor, a process that relies on several stringent assumptions due to the complexity of the Lissajous trajectory [30–33]. Key assumptions include maintaining a minimal frequency difference between the 2 axes (1 Hz) [23,24] or adhering to specific frequency ratios [34]. To optimize the fill factor, researchers have employed exhaustive methods, such as those proposed by Hwang et al. [35,36], who developed a high frame rate frequency selection method for Lissajous trajectories. In 2020, we introduced new design rules and a fill factor calculation method that theoretically addressed the challenge of optimal trajectory design [13]. Additionally, we developed a universal Cartesian space point cloud sampling model [37], enabling image resolution to approach the theoretical limits of trajectory resolution.

Despite these advancements, the intrinsic issues of central sparsity and edge density in Lissajous trajectories, coupled with the growing demands for higher image quality, have highlighted the limitations of traditional fill factor methods. These methods, although theoretically robust, often fall short in addressing the practical challenges posed by the Lissajous scanning pattern, particularly in high-resolution imaging applications.

To meet the increasing demands for enhanced image quality, researchers have explored methods such as closed-loop control [38], dynamic analysis models [39], and nonlinear corrections to extend the frequency bandwidth of MEMS devices [40]. However, these approaches often fall short of simultaneously optimizing performance across both axes. In imaging, the region of interest (ROI) denotes areas containing more critical information, making the selection and optimization of the scanning trajectory essential for capturing detailed and high-quality images. The industry frequently employs vertical mirror stitching techniques to enhance resolution, particularly at ROI junctions.

In 2021, researchers at the Vienna University of Technology advanced the angular resolution within the ROI under Lissajous scanning mode using a phase modulation method, achieving adaptive resolution effects [41]. However, their constrained analytical model requires further refinement. Subsequent efforts have primarily focused on deriving formulas for the central area of the ROI under specific conditions [42], leaving the broader challenge of optimizing Lissajous trajectories for ROI enhancement unresolved.

Inspired by the human visual system’s gaze mechanism, this study proposes a novel method to enhance MEMS scanning systems for high-resolution imaging. By dynamically adjusting scanning trajectories based on ROIs and introducing advanced parameter modulation, the method markedly improves angular resolution and point cloud density in target areas. It effectively mitigates the limitations of traditional Lissajous scanning—particularly the sparse sampling in central regions—achieving localized density gains of up to 650%. Integrated with random modulation continuous wave (RMCW) ranging, the system yields substantial improvements in scanning accuracy and image quality, demonstrating strong potential for LiDAR and projection display applications.

The remainder of this paper is organized as follows: Results presents the theoretical foundation and experimental results of the proposed scanning method in both projection and LiDAR contexts. Discussion discusses the key mechanisms contributing to point cloud enhancement and explores the broader applicability and limitations of the approach. Materials and Methods details the parameter modulation strategies and trajectory control methods employed throughout the study.

## Results

### Theoretical foundations and challenges of Lissajous-based MEMS scanning

Lissajous trajectories offer a flexible scanning strategy for MEMS systems by adjusting the frequency ratio and phase difference between orthogonal axes, while naturally aligning with the resonant properties of MEMS scanning mirrors. To effectively integrate Lissajous trajectories into scanning imaging, we propose a framework termed “scanning imaging theory”, composed of 2 core components: trajectory design and point cloud sampling.

Trajectory design concerns the construction of scanning paths that meet specific application requirements such as frame rate, field of view, and angular resolution. Point cloud sampling focuses on identifying optimal sampling points along the trajectory to support tasks such as LiDAR ranging. Theoretically, increasing the scanning frequency and number of sampling points enhances image quality. However, MEMS devices cannot support unlimited frequency increases, presenting a key constraint for real-world systems.

In our prior work, we derived mathematical expressions for fill factor and resolution, where fill factor is defined by the maximum spacing between Lissajous curve intersections, and resolution by its horizontal and vertical components. These metrics are closely correlated: higher fill factor consistency yields better resolution. For instance, achieving 720p resolution in micro-projection displays requires MEMS mirrors to resonate at over 27 kHz, while 1080p demands even higher frequencies.

To further illustrate the challenge, we previously designed a MEMS-based 3D LiDAR system targeting 0.05° × 0.05° angular resolution, a 30° field of view, and a 10-Hz frame rate. Simulations revealed that resonant frequencies of 11,450 and 180 Hz would be required—values far exceeding the capabilities of current commercial MEMS mirrors. For comparison, a 4.2-mm-diameter Mirrorcle MEMS mirror operates at only 444 Hz, and a 1-mm mirror operates at around 4.8 kHz.

To meet such performance demands, the current industrial solution involves multimirror tiling systems. At tile junctions—treated as ROIs—the resolution is locally enhanced. For example, in commercial systems like the ML-Xs, global angular resolution may be 0.2° × 0.13°, but improves to 0.15° × 0.06° within ROIs. However, this approach comes at considerable design and manufacturing costs and does not address the intrinsic limitations of MEMS fabrication technology.

From a system perspective, our practical tests with MEMS LiDAR systems reveal that hardware and software constraints typically enforce a fixed number of sampling points per frame. This can lead to inefficient resource usage, especially when scanning unimportant background regions. For example, if only the object in front of a screen is relevant, many sampling points are wasted elsewhere, degrading overall efficiency.

To address these challenges, we propose a trajectory optimization approach that maintains the original sampling method but allows lower-spec MEMS devices to achieve high resolution selectively within ROIs. By dynamically concentrating sampling density in target areas, our method enables 2× to 6× point cloud density improvements, achieving ROI-level performance comparable to high-frequency systems, without requiring hardware upgrades. This approach ensures cost-effective, high-precision scanning, fully leveraging the capabilities of existing MEMS platforms.

### ROI-oriented optimization of Lissajous scanning for angular resolution enhancement

**Fig. 1.**
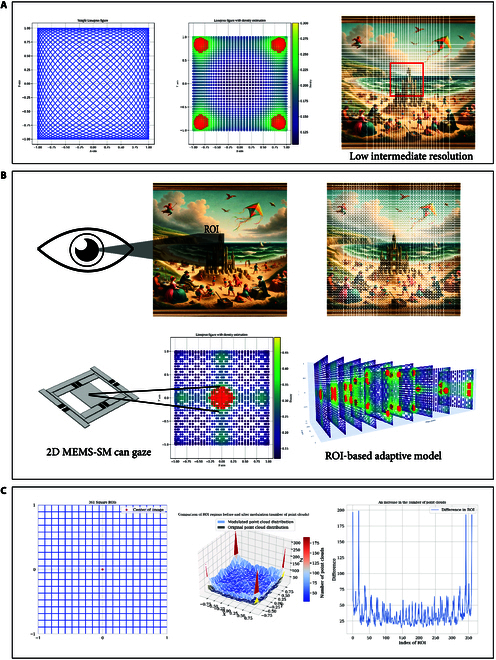
MEMS scanning mirror with “gazing” effect. (A) The Lissajous trajectory and point cloud density distribution, with denser point clouds around the edges and sparser in the middle. The red area (estimated using KDE) indicates the highest density regions. (B) Typically, the human eye focuses on the center of an image, necessitating denser sampling in the central region. Our designed method allows the MEMS scanning mirror to emulate the human eye’s gazing effect, generating denser point clouds in any area. (C) Through improvements in phase modulation methods, each area can be considered an area of interest, greatly increasing the number of point clouds in any region.

Traditional Lissajous trajectories exhibit dense sampling along the periphery and sparse coverage at the center, which limits their effective resolution in practical scanning applications. As shown in Fig. 1A, kernel density estimation highlights red regions of high sampling density—an inherent result of the trajectory’s periodicity and symmetry. For example, when the top-right region is designated as a high-density ROI, the trajectory’s symmetry inevitably increases sampling density in the diagonally opposite corners, thereby reducing the controllability and efficiency of resolution utilization.

Based on an analysis of the relationship between Lissajous characteristics and point cloud distribution, we propose 2 parameter modulation strategies to enhance resolution within the ROI. Although the 2 approaches follow distinct logical paths, both achieve notable density enhancement in the ROI without altering the overall sampling rate or requiring hardware modifications.

#### Phase modulation based on heuristic optimization

We establish a phase modulation model using a first-order Fourier series to describe the mapping relationship between the ROI position and the phase parameters of the trajectory. The Lissajous trajectory with phase modulation can be expressed as:

$$\left\{\begin{array}{c} x\left(t\right)=A\text{cos}\left(2\pi f_{x}t+\varphi_{x}\left(t\right)\right)\\ y\left(t\right)=B\;\text{cos}\left(2\pi f_{y}t+\varphi_{y}\left(t\right)\right) \end{array},\;\varphi_{x\;\text{or}\;y}\left(t\right)=a\;\text{sin}\left(2\pi \mathrm{bt}\right)\right.$$ (1)

where $f_{x}$ and $f_{y}$ are the frequencies in the horizontal and vertical directions, *A* and *B* are the amplitudes, and $\varphi_{x}$ and $\varphi_{y}$are the phase terms. The parameters *a* and *b* serve as control variables that influence the shape of the trajectory.

To systematically evaluate the impact of these parameters on scanning performance, we divided the image space into 361 ROIs (as shown in Fig. 1C). For each ROI, a heuristic search algorithm was employed to iteratively explore the values of *a* and *b*, aiming to identify the optimal combination that maximizes the number of point clouds within the target ROI.

As illustrated in the middle panel of Fig. 1C, phase modulation consistently enhances point cloud density across various ROI positions and frequency settings. Under low-frequency conditions (e.g., 26 and 25 Hz), the number of points within an ROI increased by 20 to 200, demonstrating both strong adaptability and substantial resolution improvement.

#### Amplitude modulation based on analytical modeling

To further establish the relationship between trajectory parameters and ROI positioning, we designed an amplitude modulation function, expressed as:

$$\left\{\begin{array}{c} x\left(t\right)={\mathrm{Am}}_{x}\left(t\right)\text{cos}\left(2\pi f_{x}t+\varphi_{x}\left(t\right)\right)\\ y\left(t\right)={\mathrm{Bm}}_{y}\left(t\right)\text{cos}\left(2\pi f_{y}t+\varphi_{y}\left(t\right)\right) \end{array}\;,\;m_{x\;\text{or}\;y}\left(t\right)=\left|\text{cos}\left(2\pi f_{m}t+\varphi_{m}\right)\right|\right.$$ (2)

Here, $f_{m}$ is matched to the scanning frequency of the trajectory, and $\varphi_{m}$ controls the alignment of the modulation waveform with the target ROI. The resulting modulation function is applied to the amplitude of either $x\left(t\right)$ or $y\left(t\right)$, effectively increasing point cloud density in the specified region.

In both simulation and physical experiments, the value of $f_{m}$ was set to match the actual signal frequencies of the MEMS scanning mirror along the 2 axes, while $\varphi_{m}$ was varied within the range of [0,2π]. Representative results are shown in Fig. 1B, where amplitude modulation substantially improves sampling concentration within the ROI.

### Application of trajectory optimization to projection display systems

As demand for high-quality imaging continues to grow, conventional methods of improving resolution—such as increasing the fill factor—are approaching the physical limitations of silicon-based MEMS technologies. To overcome these constraints, ROI strategies offer a promising alternative by introducing intelligent control over spatial sampling density. In this study, we apply our Lissajous trajectory optimization framework to projection display systems, enabling selective enhancement of image resolution in areas of interest.

Specifically, we replicate a projection display scenario by simulating a randomly generated grayscale image and applying an ROI-optimized Lissajous trajectory. Recognizing that the human visual system typically focuses on central image regions, our strategy allocates increased sampling density to the image center. This results in markedly enhanced angular resolution and improved visual fidelity within the ROI.

As shown in Fig. 1A and B, the optimized trajectory delivers markedly higher resolution in the central region compared to traditional Lissajous scanning under identical sampling conditions. This result validates the feasibility of modulation-based resolution enhancement in projection displays, particularly for applications requiring localized image detail without sacrificing frame rate or global field of view.

### Optimization of MEMS LiDAR technology based on the gaze model

To validate the practical applicability of our proposed trajectory optimization strategy, we implemented a gaze-inspired scanning mechanism in a MEMS-based LiDAR prototype system. The core of the system is a custom-developed MEMS piezoelectric scanning mirror with a mirror size of 3 mm × 3 mm and a scanning range of ±10°, driven in quasi-static mode without stringent *Q*-factor requirements. The 2 resonant axes operate at 190 and 365 Hz, respectively, and the device is controlled by a custom driver board paired with a high-voltage amplifier.

**Fig. 2.**
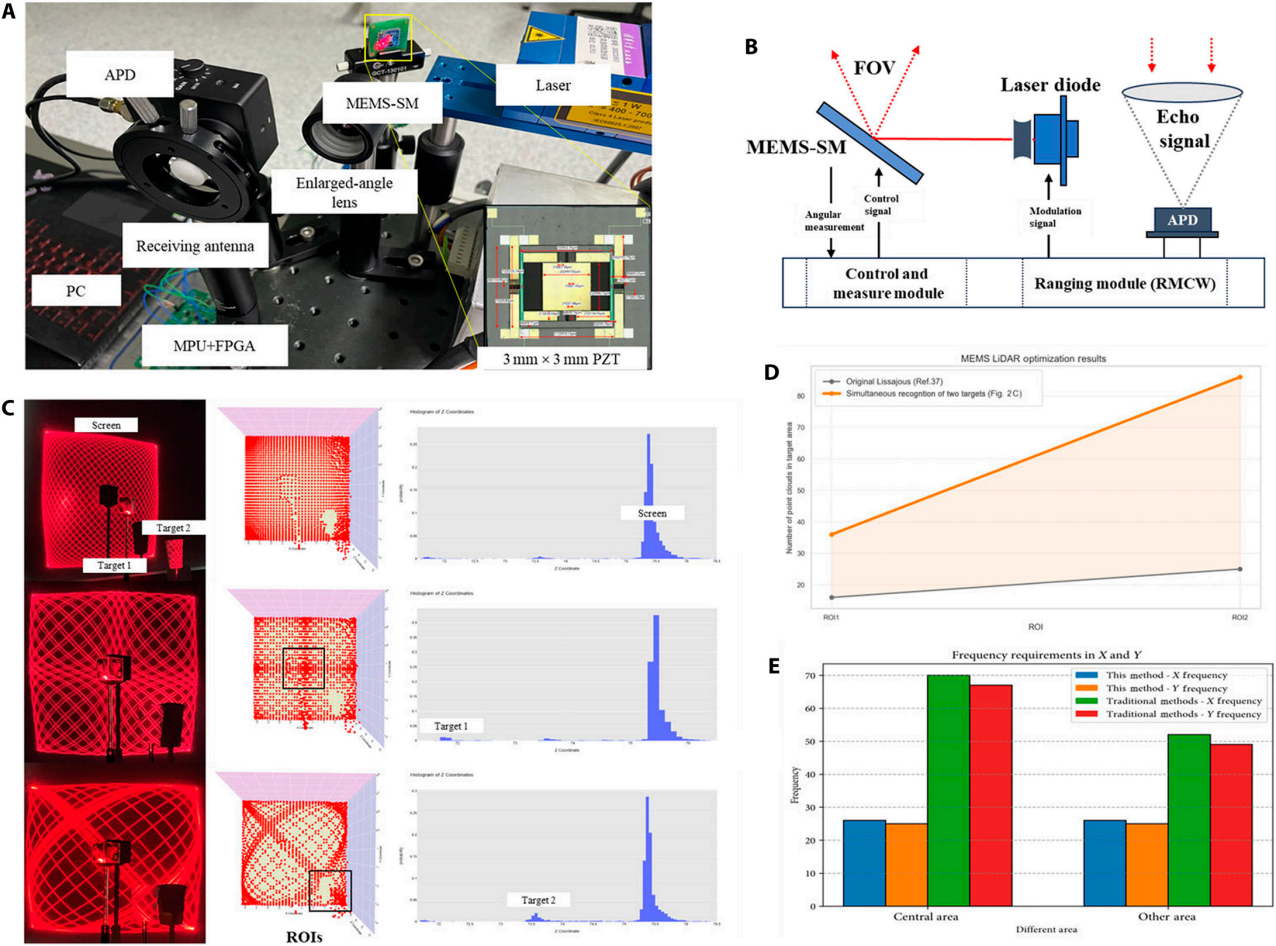
Experimental results of the gaze-based LiDAR prototype. (A) The components of the LiDAR prototype, including key elements such as the MEMS scanning mirror (MEMS-SM), controller, and detector. (B) The experimental principle of the MEMS LiDAR, primarily consisting of the ranging module and the scanning mirror driving module. (C) The specific experimental results of the multitarget LiDAR, which can selectively generate a gaze-based effect at target locations, thereby enhancing angular resolution. (D) The optimization effects for multiple targets, with the ROIs of the 2 targets achieving improvements of 650% and 244%, respectively. (E) The panel indicates that the frequency ratio required to achieve the same resolution is inversely proportional to the complexity of device design and manufacturing.

To ensure consistency with the previously established theoretical models and assumptions, imaging experiments were conducted using a MEMS LiDAR system to validate both ROI-based Lissajous trajectory optimization methods. The trajectory was modulated accordingly, with frequencies set to 26 and 25 Hz. As shown in Fig. 2C, 3 sets of experiments were performed: The first set used the original Lissajous trajectory parameters without any modulation. The second set targeted a central ROI and employed the amplitude modulation method based on Eq. 2. The modulation frequencies were matched to the 2 signal axes, and the modulation phase was set to 0. The third set focused on an off-center ROI, using the phase modulation method based on Eq. 1. In this case, only the *Y*-axis signal was modulated, with parameters *a* = 0.16 and *b* = 3.

The ranging subsystem was built on a National Instruments PXI-1082 controller with an off-axis optical configuration, which separates the transmission and reception paths to reduce echo loss caused by the limited aperture of the MEMS mirror. The system employs an RMCW technique, using a 150-MHz m-sequence as the modulation signal. A pseudo-noise (PN) code generator drives the laser module (LDM647.150.A350, maximum output power: 150 mW) to emit PAM-modulated laser pulses. The return signal is collected via a fiber optic taper and detected using a Thorlabs APD430A/M avalanche photodetector (DC-400 MHz bandwidth). An NI PXIe-5164 oscilloscope samples both the transmitted and echo signals at 1 GHz, and signal correlation is performed using a matched filter implemented in LabVIEW and Python. This configuration achieves a ranging accuracy of 2.4 cm (3σ). The system architecture is illustrated in Fig. 2B.

To validate the scanning performance, we conducted imaging experiments with 2 reflective targets: a Rubik’s cube (45 × 45 × 45 mm) and a paper cup (~50 × 70 × 50 mm), each with an approximate reflectivity of 0.8. These objects were assigned as 2 separate ROIs. At each ROI, distance measurements were repeated 10 times per sampling point, yielding a total of 2,600 point clouds.

As shown in Fig. 2C, the optimized scanning method generated dense point clouds that were accurately aligned with the positions of the 2 targets. In contrast, the unoptimized Lissajous trajectory exhibited typical distribution artifacts, characterized by dense sampling near the periphery and sparse coverage at the center.

Based on the analytical model developed in Materials and Methods, we quantified the point cloud density improvements. For Target 1 (center ROI), the point count increased from 16 to 120, representing a 650% improvement. For Target 2, the count increased from 25 to 86, corresponding to a 244% improvement. Comparable enhancements were observed across multiple other ROI locations, confirming the effectiveness and generalizability of the proposed optimization method.

Furthermore, our method enables high angular resolution (e.g., 100 points within a 0.25 × 0.25 ROI in a 1 × 1 field of view) to be achieved at lower frequencies (e.g., 30 Hz), previously only attainable at ~70 Hz. This reduces hardware complexity and signal processing demands, offering a low-frequency, high-resolution alternative for practical MEMS-SM applications.

Essentially, our approach does not alter the global sampling strategy or increase the frame rate; instead, it focuses on redistributing sampling density within the system’s fixed sampling budget. This ensures hardware feasibility while maximizing the information yield per frame.

By adaptively reallocating point clouds toward information-rich regions—without changing the total number of samples or frequencies—the system achieves a “stare-like” effect, effectively concentrating sensing attention on ROIs. This behavior mimics the mechanism of human visual attention, enabling intelligent resource allocation and resulting in substantial improvements in LiDAR imaging performance.

## Discussion

### Performance gains in angular resolution using optimized sampling

To quantitatively evaluate the improvement in angular resolution, the entire scanning field of view was normalized, and a square ROI with a side length of 0.25 was defined as the local analysis region. Due to the typical characteristics of traditional Lissajous trajectories—dense sampling at the edges and sparse sampling at the center—the angular resolution is commonly defined by the height of the largest inscribed diamond at the center. Under a frequency pair of 26 and 25, the optimal point cloud density distribution yields angular resolutions of ${\mathrm{Ar}}_{x}=0.0602\;{}^{\circ}$ and ${\mathrm{Ar}}_{y}=0.0626\;{}^{\circ}$. With our optimization method, the angular resolution within the central ROI was further improved to a maximum of 0.009°, as illustrated in Fig. 2C.

This enhancement holds across other frequency combinations as well. We conducted a comprehensive analysis of all Lissajous frequency pairs in the range of 10 to 120, where the greatest common divisor (GCD) equals 1—covering the majority of practically feasible configurations. The improvement in angular resolution is quantified as the relative increase with respect to the original value, computed using:

$$\text{Improvement Gain Ratio}=\frac{A_{\text{optimized}}-A_{\text{original}}}{A_{\text{original}}}$$ (3)

**Fig. 3.**
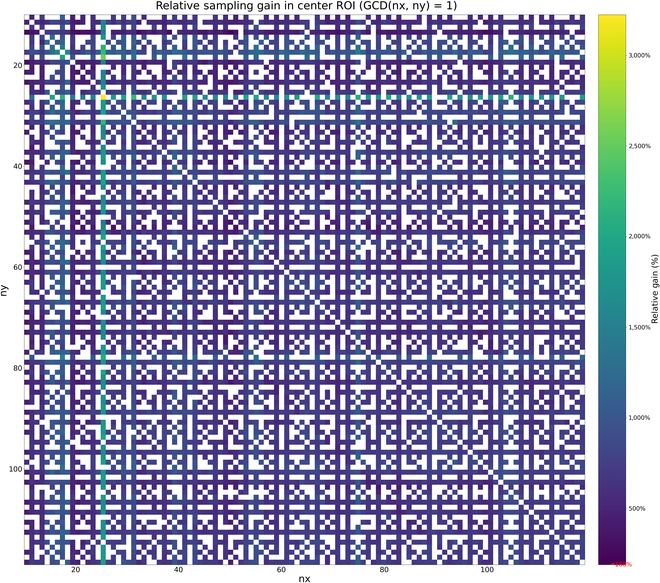
Heatmap showing the relative improvement in angular resolution across Lissajous frequency combinations (frequencies from 10 to 120, GCD = 1). All evaluated frequency pairs exhibit positive gains, with the minimum improvement reaching 188%. Darker regions indicate higher enhancement levels in ROI angular resolution.

A comparison of angular resolution before and after optimization reveals that all valid frequency pairs yielded substantial improvements (see Fig. 3), with the minimum relative gain reaching 188%.

### Parameter modulation and density enhancement mechanism for Lissajous trajectories

The proposed point cloud density enhancement strategy is fundamentally based on the targeted modulation of key parameters in the 2 periodic signals that define the Lissajous trajectory. This approach preserves the original sampling method, ensuring that the total number of point clouds remains constant, while enabling local density optimization without increasing system complexity. Given the strong correlation between point cloud distribution and frequency parameters, our optimization focuses primarily on phase and amplitude modulation.

**Fig. 4.**
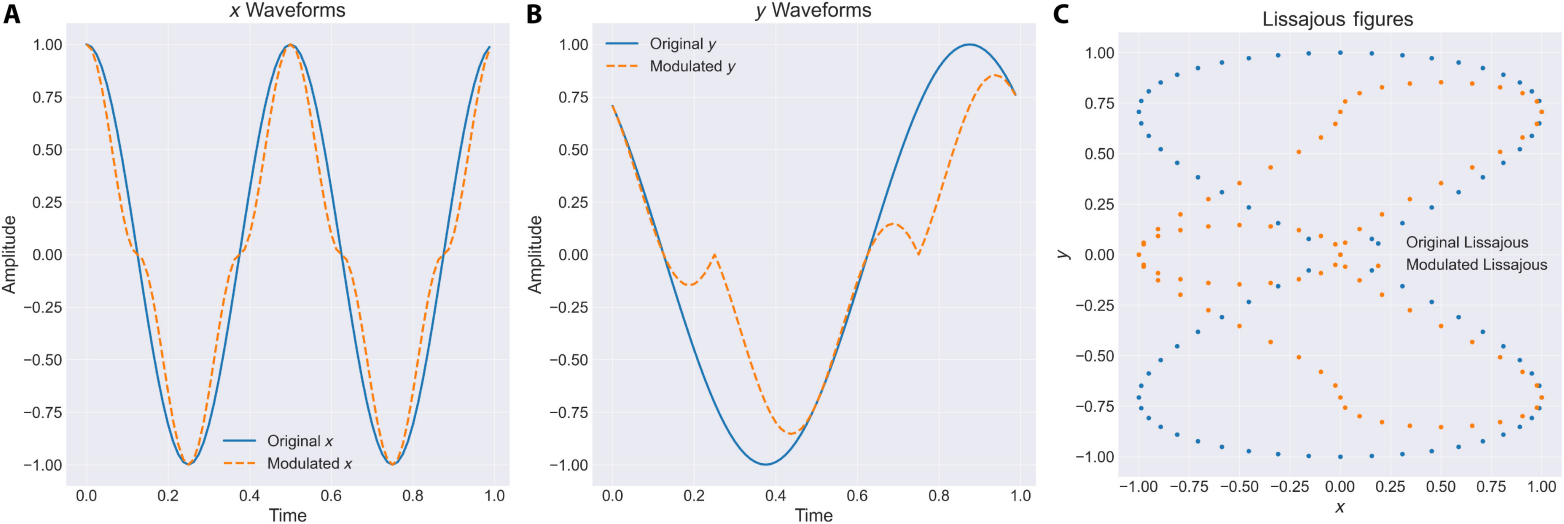
Changes in *X* and *Y* trajectories and Lissajous waveforms before and after modulation: (A) *X*-axis waveform comparison displays the changes in the waveform along the *X* direction before and after modulation, using different colors or line types to distinguish between the pre-modulation (original waveform) and post-modulation (adjusted waveform). (B) *Y*-axis waveform comparison similarly shows the waveform changes along the *Y* direction before and after modulation, visually presenting the specific effects of modulation on the waveform. (C) Lissajous trajectory comparison contrasts the Lissajous trajectories before and after modulation, with either overlapping or side-by-side displays to highlight the changes brought about by modulation.

The first method—phase modulation—adjusts the phase of the scanning signals over time, allowing for fine-tuned shifts along one axis to concentrate sampling points within a specified ROI. However, this technique has limited improvement capacity and lacks a straightforward theoretical model for parameter determination (see Fig. 4A).

In contrast, the second method—amplitude modulation based on analytical modeling—offers a more direct and interpretable mechanism. By strategically adjusting the signal amplitude, the dwell time of the trajectory within the ROI is effectively extended, leading to a substantial increase in point cloud density in the target region (see Fig. 4B and C). Analytical results further demonstrate a clear linear relationship between amplitude modulation parameters and ROI spatial location, providing a solid theoretical basis for parameter selection.

### Limitations, applicability, and future prospects of gaze-based Lissajous scanning

The gaze-based Lissajous scanning method greatly enhances point cloud density and angular resolution within ROIs. However, its effectiveness depends on precise modulation control, making it better suited for targeted scanning rather than uniform full-field coverage. Due to the inherent symmetry of Lissajous trajectories, boosting density in one ROI may inadvertently affect other regions unless carefully calibrated. Additionally, reduced sampling outside the ROI may limit its applicability in tasks requiring global scene detail.

Despite these limitations, the method is well-suited for applications with localized resolution demands, such as object detection in autonomous driving, ROI-focused projection displays, and surveillance of critical zones. It achieves substantial resolution gains without increasing hardware complexity or sampling frequency.

In the future, integrating this approach with real-time ROI detection (e.g., vision-based algorithms) could enable adaptive modulation based on scene understanding. Hybrid scanning strategies or multisensor coordination may also expand its effectiveness across diverse environments.

## Materials and Methods

### Analytical model for point cloud distribution under Lissajous trajectories

The parametric expressions for Lissajous trajectories are represented orthogonally by cosine functions as follows:

$$\left\{\begin{array}{c} x\left(t\right)=A\text{cos}\left(2\pi f_{x}t+\varphi_{x}\right)\\ y\left(t\right)=B\text{cos}\left(2\pi f_{y}t+\varphi_{y}\right) \end{array}\right.$$ (4)

where $f_{x}$ and $f_{y}$ are the frequencies in the horizontal and vertical directions, *A* and *B* are the amplitudes, and $\varphi_{x}$ and $\varphi_{y}$ are the phase terms.

The parametric expression defining a ROI in an image is given by:

$$\mathrm{ROI}=\left(x_{r},\;y_{r},\;w,\;h\right)$$ (5)

where $x_{r}$ and $y_{r}$ represent the central coordinates of the ROI, and w and h represent the width and height of the area, respectively. For simplicity and ease of calculation, this ROI is set as a square, i.e., w=h=d.

Within any single cycle of these trigonometric functions, it is established that:

$$\forall X\in \left[x_{1},\;x_{2}\right],\exists \left[t_{1},\;t_{2}\right],\left[t_{3},\;t_{4}\right]$$ (6)

The conditions for intersection and duration within the ROI are captured by 2 intervals of solutions, depending on the segment of the trigonometric function active during sampling. Equation 5 corresponds to 2 time intervals of solutions. When situated in the part of the trigonometric function where $X^{\prime }<0$, the time expressions for the ROI are:

$$\left\{\begin{array}{c} t_{1}^{\prime }=\text{arccos}\left(x_{r}+\frac{d}{2}\right)\times \frac{1}{2\pi f_{x}}\\ t_{2}^{\prime }=\text{arccos}\left(x_{r}-\frac{d}{2}\right)\times \frac{1}{2\pi f_{x}} \end{array}\right.$$ (7)

When in the trigonometric function where $X^{\prime }>0$, the other set of time expressions is:

$$\left\{\begin{array}{c} t_{3}^{\prime }=2\pi -t_{1}^{\prime }\\ t_{4}^{\prime }=2\pi -t_{2}^{\prime } \end{array}\right.$$ (8)

The forms and conditions for $y_{e}$ and $y_{e}$ are as follows:

$$y_{s}=\left\{\begin{array}{c} \text{max}\left[y\left(t_{1}^{\prime }\right),\;y_{r}-\frac{d}{2}\right],\left(Y^{\prime }>0\right)\\ \text{min}\left[y\left(t_{1}^{\prime }\right),\;y_{r}+\frac{d}{2}\right],\left(Y^{\prime }<0\right) \end{array}\right.$$ (9)

$$y_{e}=\left\{\begin{array}{c} \text{max}\left[y\left(t_{2}^{\prime }\right),\;y_{r}-\frac{d}{2}\right],\left(Y^{\prime }<0\right)\\ \text{min}\left[y\left(t_{2}^{\prime }\right),\;y_{r}+\frac{d}{2}\right],\left(Y^{\prime }>0\right) \end{array}\right.$$ (10)

Given a sampling interval $\Delta t=\frac{1}{4n_{x}n_{y}}$, the number of point clouds n within a single cycle can be calculated by:

$$n=\left|\left[\frac{\text{arccos}\left(y_{s}\right)}{\Delta t}\right]-\left[\frac{\text{arccos}\left(y_{e}\right)}{\Delta t}\right]\right|+1\;\left(\text{or}\;0\right)$$ (11)

The value 1 is taken when the ROI boundary and point cloud sampling endpoints coincide. Since the formula is primarily based on parameters derived from the *X*-direction, the total number of point clouds $n_{\text{total}}$ only needs to consider the cumulative frequency along the *X*-direction:

$$n_{\text{total}}=\sum_{i=1}^{n_{x}}n_{i}$$ (12)

### High-accuracy and high-sensitivity ranging strategy for MEMS-based LiDAR systems

In the MEMS LiDAR prototype, the ranging method is based on our previously developed absolute distance measurement approach [43]. On the transmitting side, a spread-spectrum code structure was designed to modulate the laser pulses. On the receiving side, a delay-locked loop optimized for the noise characteristics of the avalanche photodiode detector was implemented to enable high-precision code phase measurement. To further enhance accuracy, code loop filtering and narrow correlation techniques were employed.

The system demonstrates high single-point ranging accuracy across a wide range of received signal-to-noise ratios, from 13.7 to −6.3 dB. Experimental results verify that the proposed code-division multiplexed laser ranging method achieves high performance in terms of precision (2.4 cm at 3σ), interference resistance, and receiver sensitivity, with a minimum detectable signal level of −48.1 dB.

## Data Availability

Data will be made available on request.
