# Supplementary material for: Vision Gaze-Driven Micro-Electro-Mechanical Systems Light Detection and Ranging Optimization
Source: Research (Wash D C). 2025 Jun 23;8:0756. doi: 10.34133/research.0756 (PMC12185148; doi:10.34133/research.0756)
Supplement: Supplementary 1 — Figs. S1 to S4 [file research.0756.f1.docx]

Supplementary Material

**Vision Gaze-Driven MEMS LiDAR Optimization**

Supplementary Figures S1 to S4

These materials aim to enhance the clarity and reproducibility of our method, as well as to address specific questions raised during peer review.

In our system, while regions of interest (ROI) are prioritized for enhanced resolution, peripheral areas are still monitored to ensure system safety and context awareness. However, fixed sampling rates in conventional MEMS-based LiDAR systems often lead to inefficient data utilization. This inefficiency becomes particularly evident when a large number of sampling points are unnecessarily distributed over irrelevant background structures.

Our approach improves this by concentrating high-resolution scanning only within ROIs and minimizing redundant sampling outside these areas, thereby optimizing resource usage without requiring hardware upgrades.


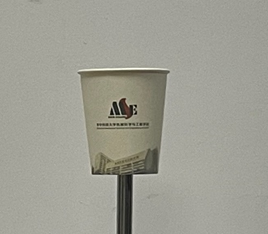

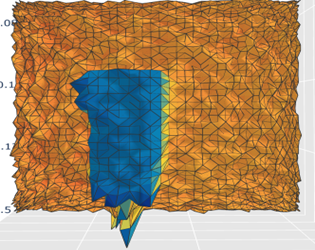


sFig. 1. LiDAR experiment result: a large number of sampling points are wasted on the back wall.

A key innovation in our method is the use of amplitude modulation to adjust the Lissajous scanning trajectory. This enables trajectory reshaping while keeping the MEMS mirror operating at its resonant frequency, enhancing stability and control accuracy.

To aid understanding, we provide a simplified example: with the drive frequency fixed at 26 Hz on both axes, we vary the modulation amplitude and phase independently. This demonstrates a linear relationship between amplitude settings and the resulting scan coverage.


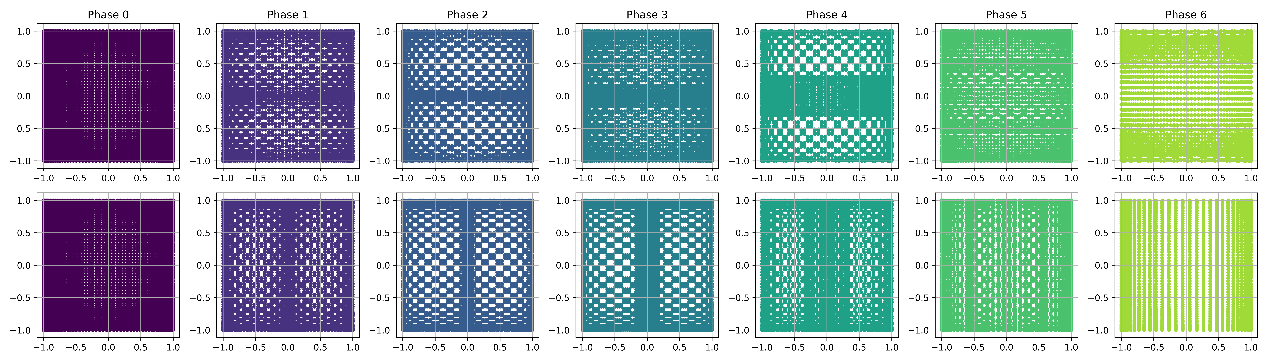


sFig. 2. Amplitude modulation method: linear variation of scanning trajectory.

While the original manuscript described the modulation method conceptually, the improved version includes visual comparisons between the conventional point cloud distribution and our ROI-enhanced result.

The scanning area was divided into a 19×19 grid (361 ROIs), with modulation parameters adjusted accordingly to concentrate sampling density where needed. This strategy mimics human visual attention, increasing point cloud density in critical areas while reducing it elsewhere.


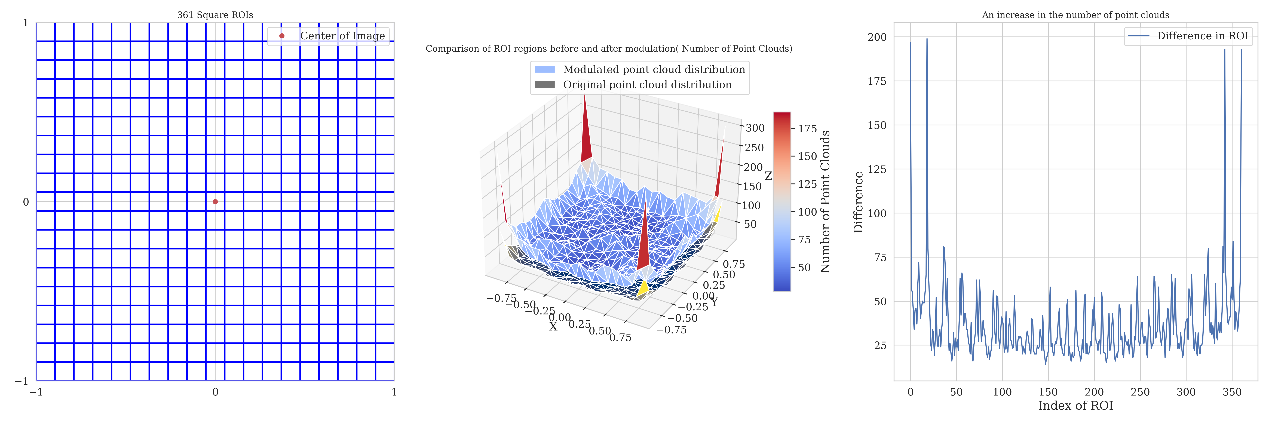


sFig. 3. ROI enhancement using parameter modulation methods.

For completeness, the prototype system architecture is provided below. It integrates the MEMS scanning mirror, laser modulation unit, control circuit, and data acquisition system. The full system supports synchronized amplitude-phase modulation and real-time ROI-targeted scanning.


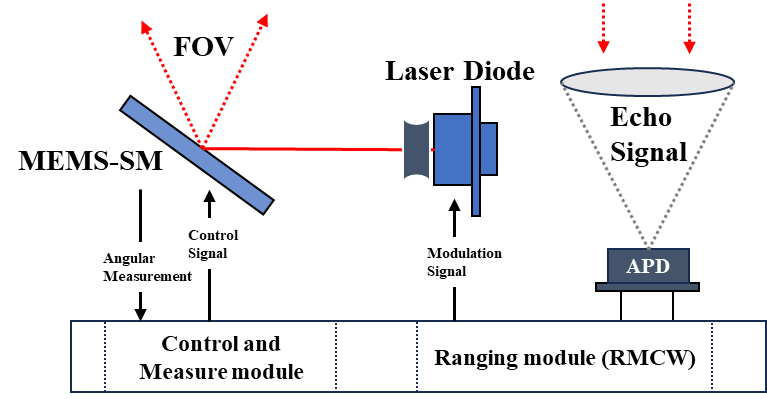


sFig. 4. Overall system architecture of the MEMS-based LiDAR prototype.
